# Menstrual irregularities associated with COVID-19 vaccines among women in Saudi Arabia: A survey during 2022

**DOI:** 10.1515/med-2023-0804

**Published:** 2023-10-09

**Authors:** Mohamed Salih Mahfouz, Maha Murtada Abdelmageed, Ahmad Y. Alqassim, Taif Khalid Mohammed Hakami, Maryam Mohammed Alshekh, Dalal Mohsen Ali Hamithi, Fatma Dia Haidar Alakhdar, Norah Mohammed Ayyashi, Ryof Mousa Ahmad Madkhali

**Affiliations:** Family and Community Medicine Department, Faculty of Medicine, Jazan University, Jazan 45142, Saudi Arabia; Obstetrics and Gynecology Department, Faculty of Medicine, Jazan University, Jazan 45142, Saudi Arabia; Faculty of Medicine, Jazan University, Jazan 45142, Saudi Arabia

**Keywords:** COVID-19, vaccine, menstrual disturbance, Jazan

## Abstract

Some changes appeared in women’s menstrual cycle after receiving the coronavirus disease 2019 (COVID-19) vaccine, but the information about the pattern and characteristics of these symptoms was unclear. This study was conducted to estimate the prevalence of menstruation change and evaluate the association between COVID-19 vaccination and the occurrence of such disturbance. An online web-based survey was conducted during March–April 2022 that targeted 729 COVID-19 vaccinated women aged between 18 and 45 years in the Jazan region of Kingdom of Saudi Arabia. The tool collected demographic information, psychological data, and COVID-19 post-vaccination side effects. The overall prevalence of menstrual change among the women was 60.9% (95% CI 57.3–64.4). 66.3% and 64.1% of women, respectively, in the age group of 25–34 and 35–45 years were more affected. Most of the detected abnormalities were related to delayed menstruation and changes in pain intensity. Menstrual disturbances that occur after immunization are transient and have no long-term implications. Menstrual disorders are prevalent before vaccination, but there is a considerable increase following vaccination. Because there is no apparent cause for these post-vaccine disturbances, and their effects are difficult to anticipate, it is preferable to warn those concerned and encourage them to learn more about the biological changes causing these problems.

## 1 Introduction

The coronavirus disease 2019 (COVID-19) outbreak emerged as a serious pandemic, murdering over 4 million people worldwide and causing huge global health concerns [1,2]. So far, in the twenty-first century, humans have gone through three fatal pandemics related to emerging coronaviruses: severe acute respiratory syndrome (SARS), Middle East respiratory disease (MERS), and COVID-19. All viruses that cause acute respiratory tract infections are highly contagious and have resulted in significant mortality rates [3]. COVID-19 is diverse from both SARS-CoV and MERS-CoV [4]. COVID-19 can adapt to a new environment through mutations and are designed to modify host tropism; consequently, the risks are ongoing and long-term [4,5,6]. There was an urgent need for safe and effective prophylactic vaccines to contain the epidemic, which had severe medical, social, and economic consequences [7].

Clinical studies produced some types of vaccination that have proven to be more than 95% effective, such as Pfizer-BioNTech, Oxford University-AstraZeneca, Moderna/NIH, and Johnson & Johnson (J&J/Janssen) [8]. Vaccines provide a biochemical reaction that mimics the real infection to elicit an immune response [9,10]. It is extremely successful in the real-world environment by decreasing infections, hospital admissions, and deaths in the months after its introduction [11]. However, many side effects of the COVID-19 vaccine were reported by healthcare workers. The symptoms most commonly reported with the Pfizer-BioTech mRNA (BNT162b2) vaccine are soreness, fatigue, myalgia, headache, chills, fever, joint pain, nausea, muscle spasm, sweating, dizziness, flushing, feelings of relief, brain fogging, anorexia, localized swelling, decreased sleep quality, itching, tingling, diarrhea, nasal stuffiness, and palpitations in some cases [12]. While symptoms shown after getting Oxford-AstraZeneca (ChAdOx1 nCoV-19) vaccines are pain at the injection site and tenderness.

Looking back on various important historical crises, such as the 2003 SARS outbreak in Asia, gender-focused investigations and analyses are frequently ignored despite its importance [13]. The gender factor has also been ignored since the rapid spread of COVID-19, particularly in research on antibodies produced by vaccination [14,15]. As a precautionary measure, the gender implications must be taken into account by researchers and health workers during the study and development of the vaccines [16,17]. The most important gender implications that must be studied are related to women’s reproductive [18,19]. Menstrual cycle (MC) is a cyclical change that occurs periodically in the uterine endometrium. The MC length varies widely, but on average, it lasts 28 days from the start of one period to the beginning of the next. Hormones are released in a negative and positive feedback loop to organize the MC. Depending on age, occupation, and country of residence, the prevalence of irregular menstruation ranges from 5 to 35.6%. Irregular menstruation has a negative impact on women’s mental health, productivity, and work [20–24].

During the COVID-19 pandemic, especially during the vaccination campaign, many women have observed and noted changes related to the menstrual frequency, duration, regularity, and volume by a complex interplay of hormones that interact with the immune, vascular, and coagulation systems. These interactions can influence menstrual bleeding and the severity of premenstrual symptoms. Considering that women are in a unique situation to face an epidemic, changes in the menstruation observed in a large group of women who received the vaccine have not been discussed [25].

There are some concerns about whether the COVID-19 vaccine is responsible for such changes or if it is only a response to other factors in surrounding conditions that could affect the regularity and intensity of menstruation, such as stress, anxiety, inadequate nutrition, or some drugs and diseases. The main objectives of this research are to first assess if receiving either Pfizer-BioNTech mRNA (BNT16262), Oxford-AstraZeneca (ChAdOxl nCoV-19), or Moderna/NIH vaccines is associated with menstrual change among women in Jazan region, Saudi Arabia, second to evaluate the short-term side effects following receiving either Pfizer-BioNTech MRNA (BNT16262), Oxford-AstraZeneca (ChAdOx1 nCoV-19), or Moderna/NIH vaccines among women in Jazan region, Saudi Arabia, and finally to identify different factors associated with COVID-19 vaccines among women.

## 2 Methods

### 2.1 Study design, setting, and population

**Figure 1:**
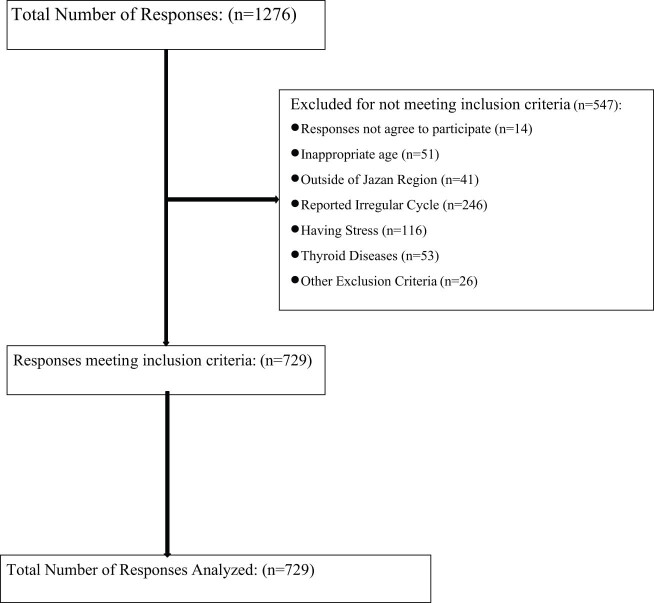
Inclusion criteria for the study.

An analytical cross-sectional study was conducted in the Jazan region. The region is one of the 13 administrative regions of Kingdom of Saudi Arabia (KSA), located in the southwest of the country. It has an area of about 13,457 km^2^ and is populated with 1,603,659 people. The study targeted women of reproductive age (18–45) who received any of the COVID-19 vaccination. Exclusion criteria involved women in the pre-pubertal or post-menopausal stage (*n* = 51), women who reported stress (*n* = 116), and women suffering from hormonal disease or any disease that may affect normal menstruation (*n* = 264). Women who used a certain medication associated with changes in normal menstruation were also excluded from the analysis (*n* = 26). A detailed description of the requirement procedure is presented in Figure 1.

### 2.2 Sampling procedures

This research’s sample size was calculated based on the statistical formula for a cross-sectional survey, which is the initial sample size = (*z* ^2^ × *p* [1 − *p*])/*d* ^2^. Since there is no estimate for the impact of the COVID-19 vaccine among women, it is safer to set the prevalence at 50%. Based on the values *p* = 0.5, *d* the desired marginal error = 0.04, *z* = 1.96, and after accounting for a 25% non-response rate, the final sample size increased to 750 women. We adopted a mixed design between random and non-random sampling to implement the survey. In the first stage, we randomly selected 5 governates of the 13 administrative units comprising the Jazan region. In the second stage, we initiated a snowball sampling in each selected administrative unit. Women were invited to join the study via the survey link.

### 2.3 Data collection method and study tool

The data were collected using an online web-based questionnaire that targeted vaccinated women between 18 and 45 years old in the Jazan region of KSA. The questionnaire for this research was designed after consulting many related research [26–29] and involved approximately 80 items. The instrument included first demographics data, including age, education, marital status, occupation, mode of living, tobacco use, weight, and height (ten questions); second, history of psychological issues, any diseases, and medications affecting normal menstruation (15 questions); third, COVID-19 vaccine uptake and post-vaccination side effects of COVID-19 like fever, tenderness at the site of injection, muscles pain, headache, lethargy (15 questions); fourth, questions on menstruation patterns before COVID-19 (20 questions); and finally, menstrual problems and their relation with COVID-19 vaccinations, which included irregular menstruation, abnormal vaginal bleeding, amenorrhea, menorrhagia, dysmenorrhea, and premenstrual symptoms, after being assessed subjectively by the participants (20 questions). Our focus is assessing the possible effect of the COVID-19 vaccine on women’s menstruation. The main outcome of this study was women with a change in cycle length (in days), the first cycle after the vaccine intake. We examined the proportion of women who experienced a significant change in cycle length, and we defined irregular menstruation as periods that occur within than 24 days or after 38 days. Face validity and internal consistency based on Cronbach’s Alpha were used to evaluate the study instrument. Experts in the field evaluated the questionnaire to assess whether the instrument’s content suited its aims. The internal consistency reliability was assessed using a small number of participants (30) through a pilot study conducted before the final implementation of the research. It produced an acceptable level of (Cronbach’s Alpha = 0.70).

### 2.4 Statistical analysis

The data analysis was performed using the IBM SPSS Statistics for Windows, Version, 26.0. Armonk NY: IBM Corp program. Data analysis involved descriptive statistics as well as inferential statistics. Simple tabulation, frequencies, and 95% confidence intervals (CI) were used to describe the prevalence of change in menstruation. The association between menstruation change as a result of COVID-19 and a set of categorical variables was assessed using the Chi-squared test. Logistic regression analysis was used to assess the predictors of menstruation change among the studied ladies. Odds ratios and their 95% CI were calculated for each separate predictor variable. A *p* value less than 0.05 was used to indicate statistical significance.

### 2.5 Ethics

We adopted the Ethical standard of the Saudi Bioethics Committee for conducting this research. Ethical approval was obtained from the Jazan University Ethics Committee with the reference number REC-43/05/091. Informed consent was obtained from all study participants. All information was kept confidential and used for research purposes only.

## 3 Results

Table 1 presents the sociodemographic and vaccine characteristic of the study participants. Most of the women were in the age group (18–24) years. The majority of the women, 583 (80.0%), had undergraduate degrees. Regarding marital status, 414 (56.8%) were single, and 294 (40.3%) were married. Employment status showed that unemployed women were 509 (69.8%). The BMI status showed that almost half of them, 331 (45.4%), are of normal weight, while only 118 (16.2%) were classified as underweight. Regarding the physical activity level, 527 (72.3%) of the participants reported that they have low physical activity, 128 (17.6%) have a moderate level of activity, and 74 (10.1%) have a high level of activity. The vaccination status showed that 275 (37.7%) of the women received two doses, and 447 (61.3%) received three doses. As for the type of vaccine received in the first dose: 563 (77.2%) reported that they received Pfizer, 157 (21.5%) received Oxford (AstraZeneca), and only 9 (1.3%) received Moderna.

**Table 1:** Background Characteristics of the women involved in the study (*n* = 729)

Characteristics		*N*	%
Age groups	18–24 years	354	48.6
	25–34 years	258	35.4
	35–45 years	117	16.0
Educational level	Primary/intermediate	10	1.4
	High school	121	16.6
	Undergraduate degree	583	80.0
	Postgraduate	15	2.0
Marital status	Single	414	56.8
	Married	294	40.3
	Divorced	15	2.1
	Widow	6	0.8
Work status	Part-time job	56	7.7
	Full-time work	164	22.5
	Not working	509	69.8
BMI categories	Underweight	118	16.2
	Normal	331	45.4
	Overweight	143	19.6
	Obese	74	10.2
	Not sure	63	8.6
Physical activity level	Low	527	72.3
	Moderate	128	17.6
	High	74	10.1
Any chronic condition	Yes	44	6.0
	No	685	94.0
Tobacco use	Yes	38	5.2
	No	691	94.8
Doses of COVID-19 vaccine received	Only one dose	7	1.0
	Two doses	275	37.7
	Three doses	447	61.3
Types of vaccine received in the first dose	Pfizer	563	77.2
	Oxford (AstraZeneca)	157	21.5
	Moderna	9	1.3

Table 2 shows surveyed women’s MC patterns before receiving the COVID-19 vaccine. Most women displayed normal bleeding days of approximately 3–8 days (95.7%). The bleeding is moderate in density for 71.1% and heaviest for 18.9% of women. The menstrual period between each cycle and the next is between 25 and 28 days for 29.2% of the women. Women’s pain does not continue throughout the menstrual period for 64.5%, while pain is persistent for 22.1%. About 52.4% could not go to work and do their activities, while 47.5% could do so. 64.9% of them use pain relievers. Almost 51.0% of women estimate the severity of the pain accompanying their period as 3–4 grades, while 27.7% rate it as being 1–2 grades. There were no important variations in normal MC patterns between the age groups of study participants (*p* > 0.05 for all).

**Table 2:** Normal pattern of menstrual cycle before receiving the vaccine among women (*n* = 729)

Normal pattern	Total	18–24 years	25–34 years	35–45 years	*p*-value	
Bleeding days	3–8	698 (95.7)	340 (48.7)	246 (35.2)	112 (16.1)	0.783
	9–20	31 (4.3)	18 (58.1)	10 (32.2)	3 (9.7)	
Bleeding density	Light	45 (6.2)	17 (37.8)	23 (51.1)	5 (11.1)	0.123
	Moderate	518 (71.1)	248 (47.9)	181 (34.9)	89 (17.2)	
	Heavy	138 (18.9)	70 (50.7)	47 (34.1)	21 (15.2)	
	Not sure	28 (3.8)	19 (67.9)	7 (25.0)	2 (7.1)	
The period between one menstrual cycle and the next	Less than 22 days	83 (11.4)	31 (37.3)	31 (37.3)	21 (25.3)	0.018
	22–24	196 (26.9)	89 (45.4)	71 (36.2)	36 (18.4)	
	25–28	213 (29.2)	99 (46.5)	76 (35.7)	38 (17.8)	
	29–32	101 (13.8)	56 (55.4)	33 (32.7)	12 (11.9)	
	33–35	25 (3.4)	13 (52.0)	9 (36.0)	3 (12.0)	
	More than 35 days	2 (0.3)	0 (0.0)	1 (50.0)	1 (50.0)	
	Not sure	109 (15.0)	66 (60.6)	37 (33.9)	6 (5.5)	
Persistence of the pain throughout the menstrual bleeding	Yes	161 (22.1)	82 (50.9)	52 (32.3)	27 (16.8)	0.045
	No	470 (64.5)	227 (48.3)	179 (38.1)	64 (13.6)	
	Not sure	98 (13.4)	45 (45.9)	27 (27.6)	26 (26.5)	
The Ability to go to work or complete the normal daily activities	Yes	347 (47.5)	195 (56.2)	125 (36.0)	27 (7.8)	<0.001
	No	382 (52.4)	160 (41.9)	133 (34.8)	89 (23.3)	
Using pain relievers	Yes	473 (64.9)	244 (51.6)	167 (35.3)	62 (13.1)	0.008
	No	256 (35.1)	110 (43.0)	91 (35.5)	55 (21.5)	
The severity of the pain accompanying the period	0	29 (4.0)	10 (34.5)	10 (34.5)	9 (31.0)	<0.001
	1–2	202 (27.7)	75 (37.1)	80 (39.6)	47 (23.3)	
	3–4	372 (51.0)	188 (50.5)	130 (34.9)	54 (14.6)	
	5–6	126 (17.3)	81 (64.3)	38 (30.1)	7 (5.6)	

Table 3 shows the prevalence of reported MC changes after receiving the vaccine among the study participants. The overall prevalence of menstrual change among the women was 60.9% (95% CI 57.3–64.4). The table further documented a constant pattern of menstruation change among the study participants (*p* > 0.05), except for age and educational level (*p* < 0.05). Regarding the type of vaccine, 60.6% of those who took Pfizer, 61.1% of those who took Oxford (AstraZeneca), and 77.8% of those who took the Moderna vaccine reported MC changes.

**Table 3:** Prevalence of reported MC changes after receiving the vaccine among women (*n* = 729)

		Women with response Yes				*p*-value
		*N*	%	95% CI		
Characteristics				Lower	Upper	
Age groups	18–24 years	198	55.9	50.7	61.0	0.026
	25–34 years	171	66.3	60.4	71.8	
	35–45 years	75	64.1	55.2	72.4	
Educational level	Primary/intermediate	4	40.0	15.3	69.6	0.003
	High School	65	53.7	44.8	62.4	
	Undergraduate degree	371	63.6	59.7	67.5	
	Postgraduate	4	26.7	9.7	51.7	
Marital status	Single	244	58.9	54.1	63.6	0.353
	Married	184	62.6	57.0	68.0	
	Divorced	11	73.3	48.3	90.3	
	Widow	5	83.3	44.2	98.1	
Work status	Part-time job	37	66.1	53.1	77.4	0.556
	Full-time work	103	62.8	55.2	69.9	
	Not working	304	59.7	55.4	63.9	
BMI categories	Underweight	73	61.9	52.9	70.3	0.661
	Normal	200	60.4	55.1	65.6	
	Overweight	95	66.4	58.4	73.8	
	Obese	47	63.5	52.2	73.8	
Physical activity level	Low	312	59.2	55.0	63.3	0.279
	Moderate	82	64.1	55.5	72.0	
	High	50	67.6	56.4	77.4	
Any chronic condition	Yes	31	70.5	56.0	82.3	0.181
	No	413	60.3	56.6	63.9	
Tobacco use	Yes	22	57.9	42.1	72.5	0.696
	No	422	61.1	57.4	64.7	
Doses of COVID-19 vaccine received	Only one dose	3	42.9	13.9	76.5	0.505
	Two doses	172	62.5	56.7	68.1	
	Three doses	269	60.2	55.6	64.6	
Types of vaccine received in the first dose	Pfizer	341	60.6	56.5	64.5	0.575
	Oxford (AstraZeneca)	96	61.1	53.4	68.5	
	Moderna	7	77.8	45.6	95.1	
Overall Prevalence		444	60.9	57.3	64.4	

Table 4 illustrates the pattern and characteristics of MC change among study participants. According to the Table, 41.8% of the women reported delay in menstruation compared with the normal pattern, while 22.9% reported early menstruation. Approximately one-quarter (23.7%) of the women stated that the bleeding density became less dense and lumpy. Those who did not notice any changes in the normal pattern of bleeding days were 64.5%. Women who noticed changes in menstrual pain intensity were 38.6%. Almost 28.3% of the women stated that these changes lasted more than 2 months after receiving the vaccine. There were no significant differences in patterns and characteristics of menstruation change according to participants’ age groups for all variables in the Table (*p* > 0.05 for all), except for bleeding density and period of changes (*p* < 0.05 for both).

**Table 4:** Pattern and characteristics of MC change among women (*n* = 729)

Characteristics			All women	18–24 years	25–34 years	35–45 years	*p*-value
Changes in the period between one menstrual cycle and the next	Delay	Yes, for a week	148 (20.3)	72 (48.6)	50 (33.8)	26 (17.6)	0.543
		Yes, for 2 weeks	65 (8.9)	36 (55.4)	22 (33.8)	7 (10.8)	
		Yes, for 3 weeks	16 (2.2)	10 (62.5)	4 (25.0)	2 (12.5)	
		Yes, for a month	38 (5.2)	18 (47.4)	16 (42.1)	4 (10.5)	
		Yes, for more than a month	38 (5.2)	13 (34.2)	15 (39.5)	10 (26.3)	
		No, there was no noticeable delay	424 (58.2)	205 (48.3)	151 (35.6)	68 (16.1)	
	Early	By a week	126 (17.3)	51 (40.5)	53 (42.1)	22 (17.4)	0.158
		By 3 weeks	41 (5.6)	19 (46.3)	12 (29.3)	10 (24.4)	
		No change	562 (77.1)	284 (50.5)	193 (34.4)	85 (15.1)	
Changes in bleeding density	It became denser and lumpier		164 (22.5)	67 (40.9)	62 (37.8)	35 (21.3)	<0.001
	It became less dense and lumpy		173 (23.7)	73(42.2)	76(43.9)	24(13.9)	
	No changes		224 (30.7)	110 (49.1)	74 (33.0)	40 (17.9)	
	Not sure		168 (23.1)	104 (61.9)	46 (27.4)	18 (10.7)	
Changes in the normal pattern of bleeding days	Yes		148 (20.3)	55 (37.2)	60 (40.5)	33 (22.3)	0.003
	No		470 (64.5)	232 (49.4)	164 (34.9)	74 (15.7)	
	Not sure		111 (15.2)	67 (60.4)	34 (30.6)	10 (9.0)	
Persistence of pain throughout the menstrual bleeding	Yes		163 (22.4)	79 (48.5)	50 (30.7)	34 (20.8)	0.183
	No		566 (77.6)	275 (48.6)	208 (36.7)	83 (14.7)	
Using pain relievers	Yes		378 (51.8)	185 (48.9)	138 (36.5)	55 (14.6)	0.184
	No		351 (48.2)	169 (48.1)	120 (34.2)	62 (17.7)	
Changes in premenstrual syndrome	Pain below the umbilical and hips	Yes	380 (52.1)	201 (52.9)	130 (34.2)	49 (12.9)	0.006
		No	349 (47.9)	153 (43.8)	128 (36.7)	68 (19.5)	
	Pain in the feet and thigh	Yes	283 (38.8)	145 (51.2)	102(36.1)	36(12.7)	0.060
		No	446 (61.2)	209(46.9)	156(35.0)	81 (18.1)	
	Nausea and fatigue	Yes	331 (45.4)	167 (50.5)	115 (34.7)	49(14.8)	0.374
		No	398 (54.6)	187 (47.0)	143 (35.9)	68 (17.1)	
	Numbness of the extremities	Yes	175 (24.00)	93 (53.1)	56 (32.0)	26 (14.9)	0.468
		No	554 (76.0)	261 (47.1)	202 (36.5)	91 (16.4)	
Change in the intensity of menstrual pain than usual	Got more intense		239 (32.8)	113 (47.3)	86 (36.0)	40 (16.7)	0.360
	Became less severe		42 (5.8)	20 (47.6)	15 (35.7)	7 (16.7)	
	No change		307 (42.1)	151 (49.2)	102 (33.2)	54 (17.6)	
	Not sure		141 (19.3)	70 (49.6)	55 (39.0)	16 (11.4)	
How many months did these changes last?	Only in the first month after receiving the vaccine		100 (13.7)	51 (51.0)	32 (32.0)	17 (17.0)	0.019
	For 2 months after receiving the vaccine		63 (8.6)	30 (47.6)	27 (42.9)	6 (9.5)	
	More than 2 months after receiving the vaccine		206 (28.3)	80 (38.8)	88 (42.7)	38 (18.5)	
	Not sure		360 (49.4)	193 (53.6)	111 (30.8)	56 (15.6)	

Table 5 illustrates the short-term side effects of the COVID-19 vaccine among study participants. Women who reported mild and moderate symptoms were 41.8% and 42.7%, respectively, while only 5.1% suffered severe symptoms. Regarding other symptoms associated with the vaccine are as follows: 51.2% of women reported redness and swelling at the injection site. Muscle pain was reported by 78.7%, while 63.1% did not report coldness paresthesia of the extremities. Almost 79.4% of the women felt tiredness and fatigue following the vaccination. Approximately 47.4% reported that their sleep quality changed significantly, 54.5% reported high temperature, and two-thirds (64.9%) experienced a headache. No significant differences were reported in short-term symptoms according to the age groups of study participants (*p* > 0.05 for all).

**Table 5:** Short-term side effects of COVID-19 vaccine among women (*n* = 729)

Side effects of COVID-19 vaccine			Total	18–24 years	25–34 years	35–45 years	*p* value
Degree of the symptoms after receiving the vaccine	Mild symptoms		305 (41.8)	153 (50.2)	105 (34.4)	47 (15.4)	0.899
	Moderate symptoms		311 (42.7)	148 (47.6)	111 (35.7)	52 (16.7)	
	Severe symptoms		37 (5.1)	15 (40.5)	14 (37.8)	8 (21.7)	
	No		76 (10.4)	38 (50.0)	2 (36.8)	10 (13.2)	
Symptoms that have been observed	Redness and swelling at the injection site	Yes	373 (51.2)	181 (48.5)	128 (34.3)	64 (17.2)	0.659
		No	356 (48.8)	173 (48.6)	130 (36.5)	53 (14.9)	
	Muscle pain	Yes	574 (78.7)	276 (48.1)	207 (36.1)	91 (15.8)	0.765
		No	155 (21.3)	78 (50.3)	51 (32.9)	26 (16.8)	
	Coldness and paresthesia of the extremities	Yes	269 (36.9)	139 (51.7)	87 (32.3)	43 (16.0)	0.373
		No	460 (63.1)	215 (46.7)	171 (37.2)	74 (16.1)	
	Tiredness and fatigue	Yes	579 (79.4)	280 (48.4)	207 (35.7)	92 (15.9)	0.918
		No	150 (20.6)	74 (49.3)	51 (34.0)	25 (16.7)	
	Change in sleep quality	Yes	348 (47.7)	176 (50.6)	122 (35.0)	50 (14.4)	0.417
		No	381 (52.3)	178 (46.7)	136 (35.7)	67 (17.6)	
	High temperature	Yes	397 (54.5)	191 (48.1)	145 (36.5)	61 (15.4)	0.738
		No	332 (45.5)	163 (49.1)	113 (34.0)	56 (16.9)	
	Headache	Yes	473 (64.9)	234 (49.5)	165 (34.9)	74 (15.6)	0.792
		No	256 (35.1)	120 (46.9)	93 (36.3)	43 (16.8)	

Univariate logistic regression analysis was conducted with “reported menstrual cycle changes after receiving the vaccine” as a dependent variable and a set of predictors that were assumed to be associated with this change. The analysis revealed that women in the age group of 25–34 years were significantly affected by this change (Crude odds ratio (COR) = 1.55, 95% CI = 1.11–2.16, *p* < 0.05) than women in the age group of 18–24. Doses of the COVID-19 vaccine received were also associated with a change in menstruation (COR = 2.23 for two doses and COR = 2.01) but with no statistical significance (*p* > 0.05) (Table 6).

**Table 6:** Predictors of menstruation change among the studied women based on univariate logistic Regression analysis

		COR	95% CI		*p*-value
Variable			Lower	Upper	
Age groups (years)	18–24	REF			
	25–34	1.55	1.11	2.16	0.010
	35–45	1.41	0.91	2.17	0.121
Educational level	Primary/intermediate	REF			
	High school	1.74	0.47	6.48	0.408
	Undergraduate degree	2.63	0.73	9.41	0.138
	Postgraduate	0.55	0.10	3.00	0.486
Marital status	Single	REF			
	Married	1.17	0.86	1.58	0.328
	Divorced/widow	2.23	0.80	6.20	0.125
BMI categories	Underweight	REF			
	Normal	0.94	0.61	1.45	0.94
	Overweight	1.22	0.73	2.03	1.22
	Obese	1.07	0.59	1.96	1.07
Doses of COVID-19 vaccine received	Only one dose	REF			
	Two doses	2.23	0.49	10.15	0.301
	Three doses	2.01	0.45	9.11	0.363

Abbreviations: REF = references; COR = crude odds ratio; CI = confidence interval.

## 4 Discussion

Although menstruation is generally not static and characterized by its variable nature from month to month across women’s reproductive lifespan, there was an increase. There were increasing concerns that COVID-19 vaccines could potentially affect the MC in women. This topic was highlighted due to the increase in complaints from some women and the reluctance of others to get immunized against the COVID-19 after realizing that the vaccine might affect the MC. Initially, this was reported by The Times newspaper, and the Medicines and Healthcare Products Regulatory Agency in the United Kingdom received nearly 4,000 reports of changes in women’s MC after receiving the COVID-19 vaccine by 17 May 2021 [30]. Male (2021) argued the possibility of a relationship between menstrual changes and receiving the COVID-19 vaccine. She also urged studies to be conducted on this topic after more than 30,000 women reported changes in their menstruation after receiving the vaccine. She notes that most women who notice changes in their MC report that things are usually back to normal by the next period [25].

In a cross-sectional study, this study investigated the prevalence of several menstrual disturbances associated with COVID-19 vaccination. The study enrolled a total of 729 women from the Jazan region between the ages of 18 and 45. As per our research, 60.9% of women had menstrual changes after receiving the COVID-19 vaccination. However, these changes do not last for long-term periods, which makes us consider that this study helps reassure many women. This outcome did not differ from many reports documenting the vaccine’s relationship and temporary menstrual disturbances. Alvergne et al. reported that only 18% of women reported MC changes after the first COVID-19 vaccine, which was considered a “very common” adverse event by the international pharmacovigilance standards [31]. Research conducted in the MENA region concluded that 86.8% of cases returned to a normal pattern in less than a month of vaccination, indicating that vaccine adverse effects are self-limiting [32].

Our research also concluded that most of the observed disturbances revolved around delay in menstruation (41.8%) and changes in pain intensity (38.6%). Almost 32.8% of the ladies had more severe menstrual pain, and 5.8% became less severe. Our research documented the persistence of pain throughout the menstrual bleeding for 22.4%, changes in blood density in most of the participants (46.2%), and an increase in the number of bleeding days for 20.3%. A study by Schoep et al. reported that 53.7% of healthy premenopausal Dutch women complained of excessive bleeding, 77.3% complained of psychological problems, and 85.4% complained of menstrual cramps [33]. These complaints were explained by several factors that could be transient such as infection, weight gain, anxiety, prolonged periods of psychological or physical stress, and endocrine diseases. All these factors may lead to the activation of the hypothalamic–pituitary–gonadal axis, disrupting the regularity of hormone secretion [34,35]. Our results were consistent with a large, well-designed study conducted in the United States, including 23,754 women, which also found delays following the vaccination dose [36]. In this regard, it should be mentioned that MC irregularities may occur not only after COVID-19 vaccination but also during/after covid infection. A recent meta-analysis [37] suggests that changes in menstrual volume and length are more associated with SARS-CoV-2 infection.

We studied the changes in the MC according to the age group and found that the age group most affected by the vaccine was between 25 and 34. In general, younger people and women have higher immunological responses than older people and men [38]. As a result, they are more likely to experience more severe side effects [39]. Several studies confirm this: In the research conducted in the Czech Republic proved that in younger people and females, a history of COVID-19 infection and comorbidities were identified as risk factors for developing side effects after COVID-19 vaccination. In addition to that, young people are more likely to develop side effects for the Pfizer vaccine [40]. Vaccine doses have a minor impact on the incidence of menstrual changes, with 42.9% in the first dose, 62.5% in the second, and 60.2% in the third. A study in Italy suggested that menstrual irregularities are slightly higher (60–70%) after the second dose [41].

COVID-19 represented a new challenge for obstetricians due to the lack of scientific evidence that could help in the different domains of reproduction and complex clinical cases [42]. Moreover, clinical trials of the current COVID-19 vaccines did not collect MC outcomes postvaccine [43]. The mechanism through which the vaccines cause menstrual symptoms is unknown. However, there is a possibility that the immune reaction is responsible for such changes. Vaccines create an immunological response that can influence the hypothalamic–pituitary–ovarian axis [42,43,44]. One of the famous hypotheses is that immune activation may transiently interfere with the hypothalamic–pituitary–adrenal axis by promoting the fluctuation of sex hormones through the positive and negative feedback loop of hormones that regulate the MC. Hypothalamic‐pituitary‐ovarian is also vulnerable to stress (e.g., poor sleep, environment, and high levels of psychological and physical stress), which results in alterations in hormonal pulsation mechanisms and MC length [45]. Based on this hypothesis and according to the World Health Organization, vaccination will increase psychological and physical stress through some of its negative effects, such as injection site pain, which is the most common local side effect, and fever, fatigue, headache, muscle aches, and joint pain, which are the most common systemic side effects [46]. As a result, we could expect menstrual alterations to be more common in people with common side effects.

Our research also focused on analyzing the general symptoms associated with the immune reaction after taking the vaccine. We found that the results were similar to other studies from Iraq and Wuhan. In Iraq, among 1,012 participants, 850 (84%) were symptomatic, and 162 (16%) were asymptomatic. The most common symptoms were: fatigue (58.1%), local injection site reactions (58%), fever (57.8%), myalgia and muscle pain (46.2%), headache (40.4%), and chills (21.4%) [47]. Similarly, Wuhan’s research found that vaccination induces fever, fatigue, headache, and muscular discomfort in vaccinated people [48]. In another hypothesis, it may be due to immune-mediated vaccine-induced thrombocytopenia. Many additional vaccines, including MMR, hepatitis A and B, diphtheria-tetanus-acellular pertussis, varicella, and even influenza, have previously been related to vaccine-induced thrombocytopenia, which can cause menstrual abnormalities [49], especially with the AstraZeneca where 6 women out of 220 people tested had high D-dimer. The test determines the likelihood of a blood clot [50]. The female gender was revealed to be a significant risk factor for high D-dimer, which was predominantly detected in women aged 55 and younger [51]. Vaccine-induced thrombotic thrombocytopenia is a rare AstraZeneca occurrence pathologically identical to heparin-induced thrombocytopenia. This is the outcome of a platelet-stimulating antibody against platelet factor 4 causing immunological thrombocytopenia [52,53].

### 4.1 Strength and limitations

Numerous recommendations have been made to conduct research on the topic, as several women have reported alterations in their MCs after receiving the COVID-19 vaccine. As of December 2022, few studies have been published on the relationship between COVID-19 vaccines and changes in the MC. We conducted this research as the first study in the Jazan region and Saudi Arabia, hoping that the results will help with proper counseling and vaccine awareness among women. The research has some limitations: first, the presence of recall bias for the information that occurred in the past. Second, this research explains the possible association but does not study the inevitable cause, and there are some other influencing factors (confounders). Third, the analysis was conducted without differentiating the type of vaccine and without correlating menstrual changes with the administration of the first or second dose. As a result, it is important to interpret the findings of our study with caution. Further research is highly recommended to overcome these limitations. The observed changes were only found in a group of vaccinated women and may lead some women to decline COVID-19 vaccinations.

## 5 Conclusion

In conclusion, we documented MC irregularities among a large segment of vaccinated women. Around 35.4% of women had no changes in their MC after immunization, while the rest of the participants had these changes. The occurrence of menstrual disturbances after vaccinations, in most cases, self-resolved within 2 months, without clinically relevant consequences. Menstrual disturbances were prevalent before vaccination, but we found a significant rise in menstrual irregularities after vaccination, especially in the case of hemorrhaging greater than normal, longer duration, and pain. As previously mentioned, these findings need further research. Based on the large number of women who initiate COVID-19 vaccines, it will be necessary to understand the underlying biological mechanisms. Also, it is essential to alert healthcare professionals and women about menstrual irregularities after the COVID-19 vaccination.
